# Excision of an Anovestibular Fistula with a Normal Anus Using a Biopsy Punch

**DOI:** 10.1155/2022/4348806

**Published:** 2022-04-16

**Authors:** Murat Sanal

**Affiliations:** Medical University Innsbruck, Department of Pediatric Surgery, Austria

## Abstract

Among anorectal malformations (ARM), the isolated H type anovestibular fistula (AVF) with a normal anus is a rarity, affecting only approximately 3% of patients with anorectal malformations. AVFs are abnormal, epithelial-lined connections between the anal canal and vestibulum, and their management is a challenge for surgeons. Complete excision of the entire fistula tract is the goal of treatment, but this can often be difficult. Moreover, recurrences are not so rare. Fistulas can occur as a result of a congenital malformation, but most often have an acquired etiology. Here, we report AVF with a normal anus in a newborn. The fistula repair was performed using a biopsy punch and layered closure without overlapping suture lines, when the patient was 4 months old.

## 1. Introduction

Anorectal malformations are congenital defects where the lower end of the digestive tract, including the anus and rectum, do not develop properly.

The most widely accepted system for identifying anorectal malformations is Pena's classification. Anal stenosis or web, rectal atresia, rectoperineal fistula, and imperforate anus without fistula are common in both males and females.

Specific female defects are rectovestibular fistula and cloaca with a short or long common channel. An isolated H-type anovestibular fistula (AVF) with a normal anus is a rarity [1, 2].

Anorectal malformations occur in approximately in 1 in every 5000 live births. They are slightly more common in males (1.2 to 1). H-type ARMs, including AVF with a normal anus, are uncommon. H-type ARMs may be isolated occurrences or accompanied by other malformations, including double vagina, anal stenosis, and other renal anomalies. These malformations are categorized as a rare regional variant in the Krickenbeck classification [1, 2]. In girls, these anomalies are further classified on the basis of anatomy; lesions are considered high if the intestinal opening originates at or above the level of levator ani muscle complex. AVF is a low lesion regarding these classifications.

The management of AVF includes surgical excision of the entire fistula with or without protective colostomy. The most used procedures are the perineal approach and the antero- or postero-sagittal approach. This study presents an H-type-isolated AVF with a normal anus and is treated successfully via transvaginal excision of the fistula using a biopsy punch.

## 2. Materials and Methods

**Figure 1:**
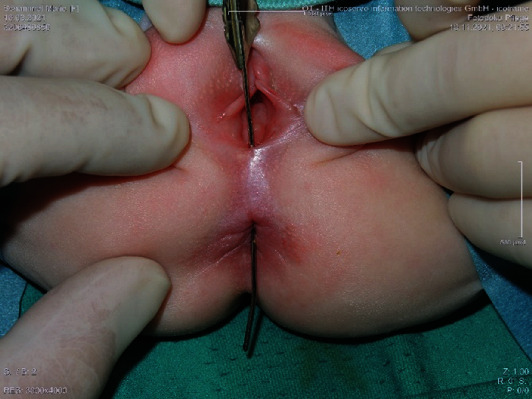
AVF (anovestibular fistula) with a normal anus.

A female newborn was referred from the pediatric department after having suffered a urinary tract infection with a suspicion of anorectal malformation. The physical examination showed stool discharge from the vestibulum. After management of the urinary infection, a detailed examination including cystoscopy, vaginoscopy, and rectoscopy was performed under general anesthesia. These showed an isolated AVF with a normal anus and no further associated anomalies. A fistula site was found below the vagina in the midline, and the internal opening was in the anterior anal wall above the dentate line (Figure 1).

To prevent recurrence of urinary tract infections, we started prophylactic oral antibiotherapy. We refrained from performing the surgery during the newborn period, instead waiting for the patient to grow. The patient was growing well; at 4 months of age and weighing 7 kg, she was scheduled for fistula repair without a colostomy.

**Figure 2:**
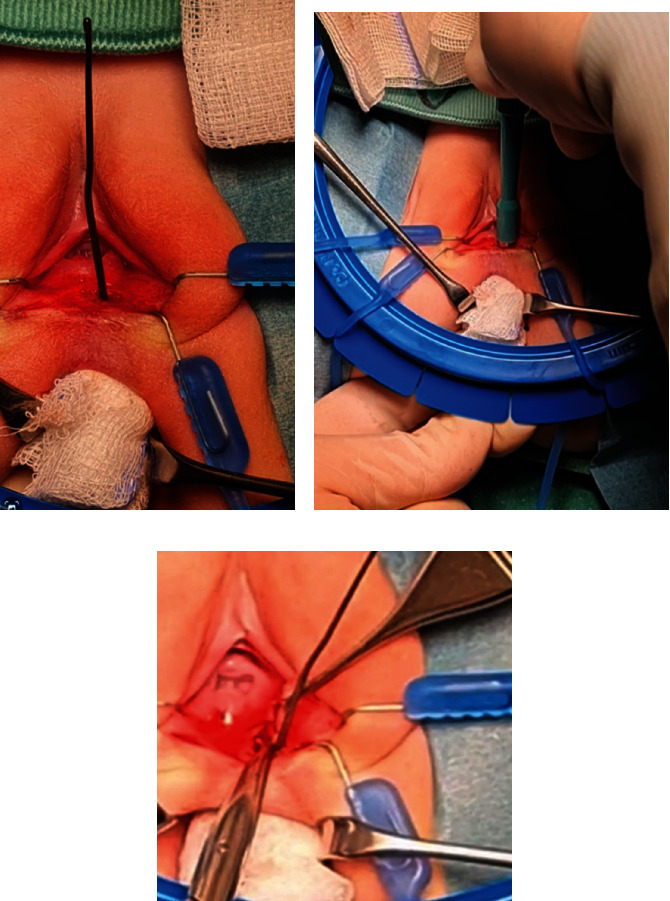
Excision of the fistula tract with a 4-mm biopsy punch.

After adequate bowel preparation, the fistulous tract was excised using a biopsy punch. A lacrimal probe with a sponge on one end was placed into the rectum and up through the vestibular fistula tract. A 4-mm biopsy punch was placed over the distal end of the lacrimal probe and down on the fistula, and with several turns, the entire fistula tract was excised (Figure 2).

**Figure 3:**
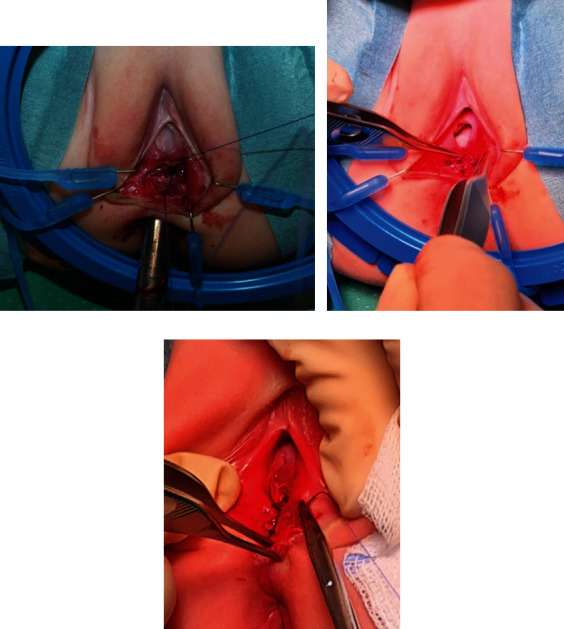
Layered closure with nonoverlapping suture lines.

After fistula excision, the lacrimal probe was removed and replaced with a Hegar rectal probe. A layered, nonoverlapping closure of the anterior wall of the anus was performed longitudinally and vestibulo-anal connective tissue transversally, and then, the vestibulum was completed with 4/0 vicryl sutures longitudinally (Figure 3). A urinary Foley catheter was placed.

Histological results of the fistula tract were reported as inflamed fibromuscular tissue and reactive epithelium. On postoperative day 3, the baby was discharged without any complications. Postoperative 6-month follow-up revealed no recurrence; the baby is healthy and in good condition.

## 3. Results and Discussion

An AVF with a normal anus can occur as a result of a congenital malformation or have an acquired etiology. In the English literature, Tahmina reports the largest series of congenital AVF with a normal anus [1]. A study by Prashant reported acquired AVF with a normal anus in 11 patients [3].

In order to make an exact diagnosis, we carried out endoscopic examinations on our patient. They showed an isolated H-Type AVF with a normal anus, without any further associated anomalies or pathological findings. The fistula site was found below the vagina, and the internal opening was in the anterior anal wall above the dentate line.

Another important topic of discussion in fistula repair is the surgical technique. There is no consensus about the surgical strategy for this type of anomaly. Some authors prefer surgical repair with a protective colostomy. Operating with a protective colostomy is clearly a safe option for healing the repaired fistula. On the other hand, every operation for protective colostomy is an additive morbidity for the patient.

Pena reported the medical records of 1700 cases and of anorectal malformations with protective colostomy. This retrospective study showed 616 complications with mislocation, stenosis, retraction, prolapsus, and general complication, and 230 of them underwent a reoperation [4].

We did not use colostomy in our case. Our decision was to perform a primary repair of the fistula, lowering the morbidity by avoiding the two colostomy operations, opening and colostomy closure. We refrained from performing the operation during the neonatal period. Only when the baby weighed a healthy 7 kg at the age of 4 months did we decide to repair the fistula without a colostomy.

Among the fistula repair procedures favored by pediatric surgeons are the perineal, antero-sagittal, and postero-sagittal approaches. They vary from simple excision of the fistula tract to extensive perineal dissection. In the English literature, the largest series with congenital AVF with normal anus belong to Tahmina, who treated 24 girls without protective colostomy via the perineal approach [1].

Fistula tract excisions are often challenging for surgeons and do need expertise and meticulous dissection and preparation to remove the fistula without any injuries.

Despite all efforts, fistula tract injuries can occur during conventional excision using scalpel, cautery, or scissors. This can result in a fragmented tissue specimen, and sometimes residual epithelial tissue is left behind, which may jeopardize the success of closure and result in recurrence of the fistula tract.

Punch biopsy excision is more safer in case of a short, small fistula outside of the sphincter mechanism, with a straightly oriented fistula tract, in contrast to conventional excision technique. The lacrimal probe functions as a guide and allows rapid, sharp, and exact removal of the fistula tract in using the punch biopsy method.

In our case, the anovestibular fistula was outside of the sphincter and was oriented straightly (Figure 1). Due to this anatomic correlation, we preferred the biopsy punch excision to remove the fistula.

The preference of the easiest method in surgery is a general opinion. And in addition to this statement, minimal handling principle should be kept in mind in pediatric surgery, especially in newborns [5]. We performed a fistula closure applying the technique of Rosenblatt, transvaginal excision using a biopsy punch, and layered closure with nonoverlapping suture lines [6]. The main advantage of this fistula repair technique is that it reduces the technical difficulty of the procedure by removing the entire fistula tract with scissors and a knife and then covering the excised region of the entire fistula tract, closing it with three layers without overlapping suture lines. The most important feature of nonoverlapping layered closure is that it reduces the possibility of recurrence.

## 4. Conclusion

As a conclusion, in the management of AVF with a normal anus, primary repair without colostomy is a good option. Our main motivation for performing the technique using a biopsy punch is that removing the entire fistula tract seems to simplify that step and the remainder of the repair, mobilizing tissue planes in order to create several nonoverlapping layers between the anus and the vestibulum.
